# Arabinoxylan-Based Particles: In Vitro Antioxidant Capacity and Cytotoxicity on a Human Colon Cell Line

**DOI:** 10.3390/medicina55070349

**Published:** 2019-07-07

**Authors:** Mayra A. Mendez-Encinas, Elizabeth Carvajal-Millan, Agustín Rascón-Chu, Humberto Astiazarán-García, Dora E. Valencia-Rivera, Francisco Brown-Bojorquez, Efrain Alday, Carlos Velazquez

**Affiliations:** 1 Biopolymers, Research Center for Food and Development (CIAD), Hermosillo, Sonora 83304, Mexico; 2 Biotechnology, Research Center for Food and Development (CIAD), Hermosillo, Sonora 83304, Mexico; 3 Nutrition, Research Center for Food and Development (CIAD), Hermosillo, Sonora 83304, Mexico; 4 Department of Chemical Biological and Agropecuary Sciences, University of Sonora, Caborca, Sonora 83621, Mexico; 5 Department of Polymers and Materials, University of Sonora, Hermosillo, Sonora 83000, Mexico; 6 Department of Chemistry-Biology, University of Sonora, Hermosillo, Sonora 83000, Mexico

**Keywords:** arabinoxylan gel, microspheres, ferulic acid, antioxidant activity, cytotoxicity, microstructure, coaxial electrospraying

## Abstract

*Background and objectives:* Arabinoxylans (AX) can gel and exhibit antioxidant capacity. Previous studies have demonstrated the potential application of AX microspheres as colon-targeted drug carriers. However, the cytotoxicity of AX gels has not been investigated so far. Therefore, the aim of the present study was to prepare AX-based particles (AXM) by coaxial electrospraying method and to investigate their antioxidant potential and cytotoxicity on human colon cells. *Materials and* *Methods:* The gelation of AX was studied by monitoring the storage (G′) and loss (G′′) moduli. The morphology of AXM was evaluated using optical and scanning electron microscopy (SEM). The in vitro antioxidant activity of AX before and after gelation was measured using the 2,2′-azino-bis(3-ethylbenzothiazoline-6-sulfonic acid) (ABTS^+^), 2,2-diphenyl-1-picrylhydrazyl (DPPH) and ferric reducing antioxidant power (FRAP) methods. In addition, the effect of AX and AXM on the proliferation of human colon cells (CCD 841 CoN) was evaluated using the 3-(4,5-dimethylthiazol-2-yl)-2,5-diphenyltetrazolium bromide (MTT) assay. *Results:* The final G′ and G′′ values for AX gels were 293 and 0.31 Pa, respectively. AXM presented spherical shape and rough surface with a three-dimensional and porous network. The swelling ratio and mesh size of AXM were 35 g water/g AX and 27 nm, respectively. Gelation decreased the antioxidant activity of AX by 61–64 %. AX and AXM did not affect proliferation or show any toxic effect on the normal human colon cell line CCD 841 CoN. *Conclusion:* The results indicate that AXM could be promising biocompatible materials with antioxidant activity.

## 1. Introduction

Polysaccharide-based carriers have become attractive materials due to their potential use in a wide range of biomedical applications. Polysaccharides exhibit interesting advantages such as biocompatibility, biodegradability, are highly safe and non-toxic [1]. Several polysaccharides, such as alginate, chitosan, guar gum, pectin, and more recently arabinoxylans (AX) have been studied for the development of novel drug delivery systems [2,3,4,5,6,7]. Particularly, AX are polysaccharides exhibiting interesting characteristics and properties that make them a promising material for its application as a drug delivery system.

**Figure 1:**
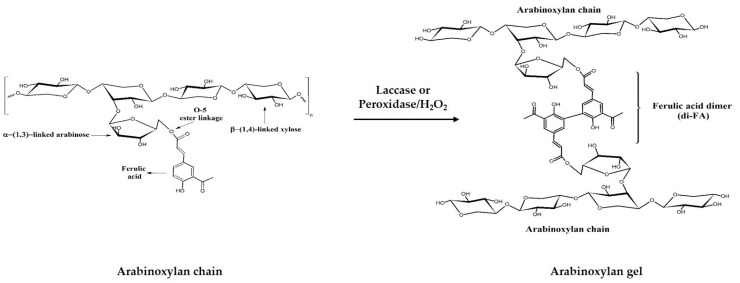
Structure of ferulated arabinoxylan and oxidative cross-linking of arabinoxylans (AX).

AX is a hemicellulose constituted by a β-(1-4)-xylopyranose backbone chain with some α-L-arabinose residues attached at O-3 and/or O-2 position [8]. In addition, ferulic acid (FA) molecules can be ester-linked to some arabinose residues at O-5 position [9]. AX has the ability to gel by the covalent cross-linking of their FA in the presence of free radical-generating agents (chemical or enzymatic) [8,10] (Figure 1). The cross-linking of AX chains leads to the formation of dimers (di-FA) and trimers (tri-FA) of FA, resulting in the three-dimensional network of the gel [11]. In addition, some non-covalent interactions such as hydrogen bonds, may be involved in the gel formation [12]. These gels are stable to pH and temperature and can absorb high amounts of water [8].

Cross-linked AX gels have been studied as matrices for the controlled release of several biomolecules (model proteins, methyl xanthine and lycopene) [11,13,14], and cells (yeast, probiotics) [15,16,17]. Moreover, the in vitro degradation of these gels by the colonic microbiota [18,19], as well as their antioxidant capacity [20] has been documented. In a previous study, laccase induced AX microspheres with antioxidant capacity were obtained by dropwise extrusion method, showing potential application as microencapsulation systems with antioxidant capacity [21]. Recently, the fabrication of core-shell AX particles for the entrapment of bifidobacteria and insulin was reported by Paz-Samaniego et al. [6]. In addition, the administration of loaded-insulin AX microspheres to diabetic rats showed a hypoglycemic effect, demonstrating its potential as oral insulin carriers [7].

The beneficial effects of AX on health are widely documented. AX exhibit antioxidant and prebiotic properties, which have been associated with the prevention of colon cancer [22]. The fermentation of AX stimulates the growth of probiotics such as *Lactobacillus* and *Bifidobacterium,* which exert positive effects on gut health [23,24]. In addition, the main end products of AX fermentation are short chain fatty acids (acetate, propionate and butyrate), metabolites with well-known positive effects on the host health. The antioxidant capacity of AX is widely related to their phenolic acid content, particularly FA, which is the most abundant in ferulated AX [25] and has been tested both in vitro and in vivo. Herrera-Balandrano et al. [25] evaluated the antioxidant capacity of AX from nixtamalized maize bran and found a positive correlation between the total phenolic acid content and the FA content with the antioxidant capacity. Moreover, a previous study showed that feruloyl oligosaccharides (FOS) showed better antioxidant activity than free FA in the inhibition of low-density lipoprotein oxidation [26]. FOS exhibited a protective effect against oxidative stress in rat plasma by decreasing the oxidative stress markers (oxidized glutathione and malondialdehyde), as well as enhancing the activity of antioxidant enzymes (superoxide dismutase, catalase and glutathione peroxidase) after ingestion of FOS [27]. On the other hand, the in vitro fermentation of AX gels appears to exert a positive effect on gut health due to its selective fermentation by gut microbiota. The cross-linking of AX limits the growth of *Bacteroides* [19], while stimulates the growth of *Bifidobacterium*. This statement was recently confirmed by in vivo studies where the administration of AX gels to induced-obese rats increased the population of *Bifidobacterium*, while *Bacteroides* decreased [28].

AX gels appear to be promising materials for their use as matrices for the delivery of bioactive molecules targeted to the colon. Moreover, their biological properties (antioxidant and prebiotic) make them more attractive materials, as they can also exert beneficial effects for host health. However, to date, there are no reports about the evaluation of the cytotoxic effect of AX gels. Furthermore, the in vitro antioxidant capacity of AX gels has been only investigated using the 2,2′-azino-bis(3-ethylbenzothiazoline-6-sulfonic acid) (ABTS^+^) method. Therefore, the aim of the present work is to prepare AX-based particles (AXM) by oxidative cross-linking in order to evaluate their antioxidant capacity using the ABTS^+^, 2,2-diphenyl-1-picrylhydrazyl (DPPH) and ferric reducing antioxidant power (FRAP) methods and cytotoxicity on a human colon cell line.

## 2. Materials and Methods

### 2.1. Materials

AX were obtained from dried distillers’ grains with solubles (DDGS) and characterized as previously described [29]. The AX presented 64% dry basis (d.b) of pure AX (sum arabinose + xylose), 5.11% glucose, a protein content of 8.2%, an A/X ratio of 1.1 and a molecular weight distribution of 209 kDa. Laccase (benzenediol: oxygen oxidoreductase, E.C.1.10.3.2) from *Trametes versicolor,* and all other chemical products were purchased from Sigma Chemical Co. (St. Louis, MO, USA).

### 2.2. Methods

#### 2.2.1. Gel Preparation

AX solutions (2% w/v) were prepared in 0.05 M citrate-phosphate buffer (pH 5.5). Laccase was used as cross-linking agent (6.7 nkat/mg AX) [29].

#### 2.2.2. Rheological Measurements

The AX gel formation was investigated by small amplitude oscillatory shear, using a strain-controlled rheometer (Discovery HR-2 rheometer, TA Instruments, New Castle, DE, USA) in oscillatory mode. AX solutions (2% w/v) were mixed with laccase (6.7 nkat/mg AX) and immediately poured onto a parallel-plate geometry (4 cm in diameter) at 4 °C. The exposed edges were sealed with silicone to avoid evaporation. The kinetic of gelation was started by a sudden increase in temperature from 4 °C to 25 °C and followed at 25 °C during 1 h. The rheological parameters monitored during gelation process were storage (G′) and loss (G′′) moduli, tan δ (G′′/G′) and crossover point (G′>G′′). Measurements were carried out at a frequency of 0.25 Hz and 5% strain. A frequency sweep was performed (0.01–10 Hz) at 5% strain and 25 °C at the end of the gel formation [29].

#### 2.2.3. Phenolic Acids

FA, di-FA, and tri-FA in AX and AX gels were quantified by RP-HPLC as previously described [30,31] after a de-esterification step. An Alltima C18 column (250 mm × 4.6 mm; Alltech Associates, Inc., Deerfield, IL, USA) and a photodiode array detector (Waters 996; Waters Corporation, Milford, MA, USA) were used. Detection was followed by UV absorbance at 320 nm.

#### 2.2.4. Preparation of AXM

**Figure 2:**
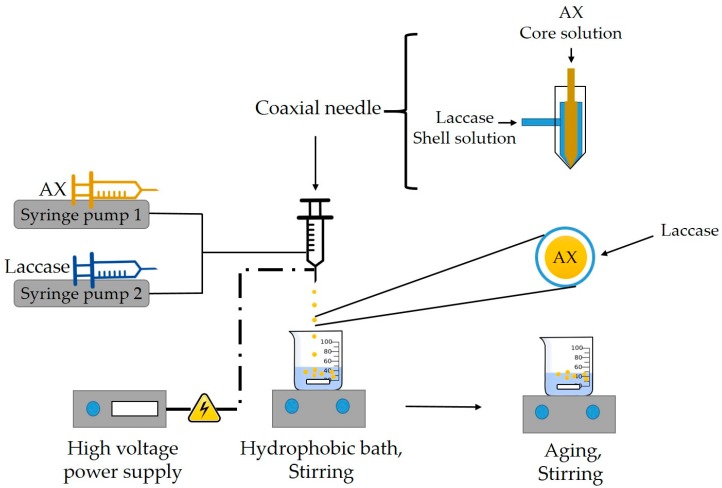
Schematic representation of preparation of AX-based particles (AXM) by coaxial electrospraying.

AXM were prepared by coaxial electrospraying method (The Spraybase^®^ electrospray device, Profector Life Sciences, Kildare, Ireland) according to Rascón-Chu et al. [32] with some modifications. Figure 2 shows a schematic representation of the coaxial electrospraying setup used to prepare the AXM. AX solution (2% w/v) in 0.05 M citrate-phosphate buffer (pH 5.5) and laccase (6.7 nkat/µL) were loaded into 1 mL plastic syringes (4 mm diameter). The stainless-steel coaxial nozzle (Spraybase^®^) consisted in two capillaries (20 G (inner) and 26 G (outer) diameter). The solutions were injected and allowed to flow through the needle at fixed flow rates of 4.0 mL/h and 0.6 mL/h for AX (shell, outer nozzle) and laccase (core, inner nozzle) solutions, respectively, using a syringe pump (WPI, AL-1000, Hitchin, UK). The distance between the needle tip and the collector was ~15 cm. A voltage of 11–13 kV was applied at the tip of the needle tip, using a high voltage power supply (Spraybase^®^ controller, Profector Life Sciences, Kildare, Ireland). AXM were collected into a hydrophobic bath under constant stirring (500 rpm) at 25 °C and aged for 12 h. The AXM were recovered by filtration, washed with ethanol:water (30:70, 50:50, 70:30 v/v), vortexed during 2 min and centrifuged (2000 *g*, 3 min, 20 °C) between each wash in order to remove the residual hydrophobic liquid. Finally, the AXM were stored at 4 °C for further analyses.

#### 2.2.5. Optical Microscopy

Morphology and size of AXM were evaluated by optical microscopy using an inverted optical microscope (Zeiss Axio Vert. A1, Carl Zeiss Microscopy, Jena, Germany) equipped with a digital camera (Axio Cam ERC 5s, Jena, Germany). A drop of AXM suspension was placed on the glass slide before observation at the appropriate magnification. The particle size of AXM was measured by image analysis (Zen 2.3 lite, Jena, Germany). The mean particle size was calculated by measuring the diameter of 120 AXM (*n* = 3). The average particle size was expressed as volume mean diameter (µm) ± SD values related to the mean [33].

#### 2.2.6. Scanning Electron Microscopy (SEM)

AXM were frozen by liquid nitrogen immersion and lyophilized in a Freezone 6 freeze drier (Labconco, Kansas, MO, USA) overnight at −37 °C/0.133 mbar. The surface morphology and microstructure of lyophilized AXM was studied by SEM (20 kV) (JEOL 5410LV, JEOL, Peabody, MA, USA). SEM images were obtained in secondary electron image (SEI) mode [34].

#### 2.2.7. Fourier Transform Infrared Spectroscopy (FT-IR)

Infrared spectra of AX or lyophilized AXM powder were recorded on a Nicolet iS50 FT-IR spectrometer (Thermo Fisher Scientific Inc., Waltham, MA, USA) in absorbance mode from 400 and 4000 cm^−1^ range at 20 °C. Thirty-two scans at a resolution of 4 cm^−1^ were averaged and referenced against air.

#### 2.2.8. Swelling Experiment

At the end of gelation, AXM were recovered by filtration, placed in a glass slide and weighed. AXM were allowed to swell as reported elsewhere [35]. The samples were blotted and weighed during 30 min. The equilibrium swelling was reached when the weight of the samples changed by no more than 3%. The swelling ratio (*q*) was calculated according to Equation (1):

(1) $$q\;=\;\frac{W_{S}\;-\;W_{AXM}}{W_{AXM}}$$

where *W_S_* is the weight of swollen AXM and *W_AXM_* is the weight of AX in the AXM. The AX weight in the AXM was calculated by considering the AX in the AX solution (2% w/v) and the fresh weight of the AXM after recovering.

#### 2.2.9. Structural Parameters

The structural parameters of AXM were estimated from the swelling measurements as previously described [35]. The molecular weight between two cross-links (*M_c_*), the cross-linking density (*ρ_c_*) and the mesh size (*ξ*) values of AXM were calculated using the model of Flory and Rehner [36] modified by Peppas and Merrill [37] for gels where the cross-links are introduced in solution. First, the *M_c_* was obtained from Equation (2):

(2) $$\frac{1}{M_{c}}-\frac{2}{M_{n}}=\frac{\left(\upsilon /V_{1}\right)\left[ln\left(1-\upsilon_{2,s}\right)+\upsilon_{2,s}+X_{1}{\left(\upsilon_{2,s}\right)}^{2}\right]}{\upsilon_{2,r}\left[{\left(\upsilon_{2,s}/\upsilon_{2,r}\right)}^{1/3}-\frac{1}{2}\left(\upsilon_{2,s}/\upsilon_{2,r}\right)\right]}$$

where *M_n_* is the number average molecular weight of AX (100,000 g/mol, considering only the xylose backbone). In equation (2), *V_1_* is the molar volume of water (18 cm^3^/g), *υ_2,r_* and υ_2,s_ are the polymer volume fractions of the gel in a relaxed state (directly after gel formation) and at equilibrium swelling, respectively. χ_1_ is the Flory polymer–solvent interaction parameter (0.5). The partial specific volume (υ) of AX was 0.4273 cm^3^/g. After *M_c_* calculation, the average mesh size (*ξ*) of AXM was obtained using Equation (3) [38]:

(3) $$\xi =\upsilon_{2,s}{}^{-1/3}{\left(\frac{2C_{n}M_{c}}{M_{r}}\right)}^{1/2}l$$

with *M_r_* representing the molecular weight of the repeating unit (xylose, 132 g/mol), *C_n_* the characteristic ratio for AX (11.5) [39], and *l* the bond length between two xyloses (0.286 nm). Finally, the cross-linking density in AXM (*ρ_c_*) was calculated from *M_c_* as previously reported by Peppas, Moynihan, and Lucht using Equation (4) [40].

(4) $$\rho_{c}=\frac{1}{\upsilon M_{c}}$$

#### 2.2.10. Antioxidant Activity

The in vitro antioxidant capacity of AX before and after gelation was measured using the ABTS^+^, DPPH and FRAP methods.

The ABTS^+^ scavenging capacity was determined as previously described [41,42,43]. The absorbance of the clear supernatant of the sample and ABTS^+^ reagent mixture was measured at 734 nm. The absorbance readings were made after the ABTS^+^ reagent was added to the sample exactly at 7, 15, and 21 min. Results were expressed as µmol of Trolox (6-hydroxy-2,5,7,8-tetramethylchoman-2-carboxylic acid) equivalent capacity per gram of sample (µmol TEAC/g). A dose-response curve of different concentrations of Trolox was performed in order to determine the antioxidant capacity of the samples.

Previously to DPPH and FRAP scavenging activity determinations, stock dispersions were prepared by dissolving AX and lyophilized AX gels in water (5 mg/mL). AX gel dispersion was homogenized using an ultrasonic homogenizer (OMNI Sonic Ruptor 400, Kennesaw, GA, USA) at 50 pulse and 30 % power for 5 min at 25 °C.

The antioxidant activity by DPPH method was measured according to the method described by Malunga and Beta [44] with some modifications. Stock dispersion was dissolved in ultrapure water to make solutions of different concentrations (0–2000 µg/mL). In addition, standard solutions of Trolox (0–15.0 µg/mL) in ultrapure water were prepared. A 45 µM DPPH work solution was prepared by mixing 1.8 mg of DPPH and 30 mL of methanol until dissolved. Subsequently, 20 mL of water was added and finally stored in the dark until used. Aliquots of 400 µL of AX solutions were mixed with 350 µL of absolute methanol. Then 750 µL of DPPH solution was added to the mixture, vortexed and left in the dark. The measurements were taken at 40 and 60 min at absorbance of 515 nm using a microplate spectrophotometer (Thermo Scientific MultiSkan Go, Madrid, Spain). Antioxidant activity was determined by means of dose-response curve of Trolox and results were expressed as µmol TEAC/g sample.

Ferric reducing ability of AX and AX gels was evaluated according to the methodology described by Benzie and Strain [45]. A working FRAP reagent was prepared reacting 10 volumes of 300 mM acetate buffer (pH 3.6), 1 volume of 40 mM 2,4,6-tri(2-pyridyl)-striazine (TPTZ) (dissolved in 40 mM HCl), and 1 volume of 20 mM ferric chloride (dissolved in water). Subsequently, 280 μL of FRAP reagent was mixed with 20 μL (5 mg/mL) of AX or AX gel dispersion, and the absorbance was read at 630 nm at a microplate spectrophotometer (Thermo Scientific Multiskan GO, Madrid, Spain) after 30 min of storage in the dark. Results were reported as µmol of Trolox equivalent antioxidant capacity (TEAC) per gram sample by means of a dose-response curve for Trolox.

#### 2.2.11. Cell Lines and Culture Conditions

The normal cell line from human colon, CCD 841 CoN (ATCC^®^ CRL1790^™^) was obtained from the American Type Culture Collection (ATCC, Manassas, VA, USA). Cell line was grown in a complete medium (D10F) that consisted of DMEM (Dulbecco’s Modified Eagle Medium) supplemented with 10% fetal bovine serum (FBS, Gibco), 1% non-essential amino acids, 100 U/mL penicillin, and 100 mg/mL streptomycin. The cells were maintained at 37 °C and 5% CO_2_ in a humidified incubator (Thermo Fischer Scientific, San Jose, CA, USA) [46].

#### 2.2.12. Cytotoxicity on Normal Human Colon Cells

The effect of AX and AXM on the proliferation of normal human colon cell line CCD 841 CoN was determined using the standard 3-(4,5-dimethylthiazol-2-yl)-2,5-diphenyltetrazolium bromide (MTT) assay [47] with some modifications [48]. In brief, cells (2 × 10^4^ cells, 50 µL) were placed in each well of a flat 96-well plate (Costar, Corning, NY, USA). After 24 h of incubation at 37 °C in an atmosphere of 5% CO_2_ to allow cell attachment, the culture medium was taken off and aliquots of D10F cell culture medium (100 µL) containing different concentrations of AX or AXM (0–1000 µg/mL) were added and incubated for 48 h. Water was used as dissolvent control and the cytotoxic drug doxorubicin was used as positive control in the assays. In the last 4 h of incubation, 10 µL of MTT solution (5 mg/ mL) were added to each well. The cell viability was assessed by the ability of metabolically active cells to reduce tetrazolium salt to colored formazan compounds. The formazan crystals formed were dissolved with acidic (0.4%) isopropyl alcohol (Sigma-Aldrich, St. Louis, MO, USA). The absorbance of the samples was measured with an ELISA plate reader (Multiskan EX, ThermoLabSystem, Shanghai, China), using a test wavelength of 570 nm and reference wavelength of 650 nm.

#### 2.2.13. Cell morphology Analysis

The effect of AX and AXM on the cell proliferation and cell morphology of CCD 841 CoN cell line was analyzed using an inverted microscope (Nikon Corporation, Tokyo, Japan). Cells were seeded at a density of 2 × 10^4^ cells/well on 96-well culture plate and incubated at 37 °C for 24 h. Different concentrations of AX or AXM (125, 250, 500, 1000 µg/mL) were added to the cells and incubated for 24 h. Cells with culture medium were used as the control. The cells were analyzed at 200× magnification [49].

#### 2.2.14. Statistical Analysis

Rheological and phenolic acids measurements were made by duplicate. The swelling experiment and cytotoxicity assay were made by triplicate. Results are expressed as mean ± SD values. Data were analyzed using one-way analysis of variance (ANOVA) with Tukey-Kramer test (NCSS, 2007, NCSS, LLC, Kaysville, TN, USA).

## 3. Results

### 3.1. Gelation and Covalent Cross-links

**Figure 3:**
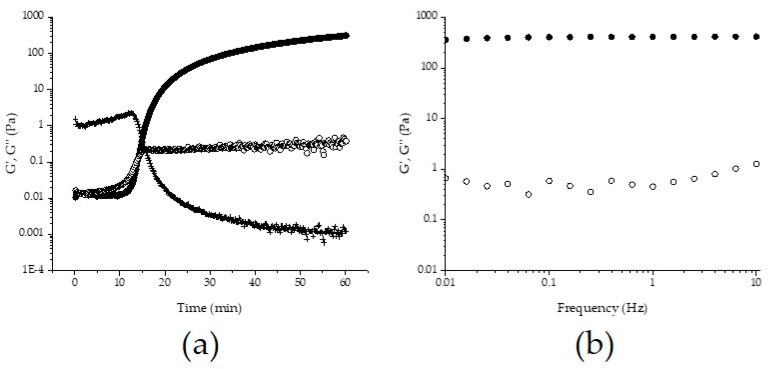
(**a**) Kinetic of gelation of AX solution (2% v/w) induced by laccase at 0.25 Hz. (**b**) Mechanical spectrum of AX gel at 1 h gelation. G′ (●), G′′ (○), tan δ (+). Measurements made at 25 °C and 5% strain.

The gelation of AX solution (2% w/v) was evaluated by small amplitude oscillatory shear following the storage (G′) and loss (G′′) moduli. The kinetic profile of AX solution showed a rapid increase in G′ and G′′ moduli at the beginning of gelation, followed by a higher increase in G′ value until reach a stability region known as *plateau* (Figure 3a). The gelation time (*t_g_*) or sol/gel transition point (G′ = G′′), calculated from the crossover of G′ and G′′ curves (G′ > G′′) was 15 min for AX gel. According to the rheological measurements, the maximum G′ and G′′ values for the AX gel at the plateau region (60 min) were 293 y 0.31 Pa, respectively (Figure 3a). The tan δ value decreased during the AX gel formation, indicating the presence of an elastic covalent system (Figure 3a). The mechanical spectrum of AX gel at 1 h gelation is presented in Figure 3b, showing a linear G′′ independent of frequency, and a G′ smaller than G′′ and dependent of frequency.

The extent of the FA monomer and the crosslinking structures (di-FA and tri-FA) were measured before and after the gelation of AX (Table 1). At the end of gelation, 35% of the initial FA content in AX was oxidized by the action of laccase leading to the formation of covalent cross-links structures. The loss of FA monomers is in accordance with the increase of the di-FA and tri-FA detected in AX gels. However, the amounts of di-FA and tri-FA recovered after gel formation represented 85% of the total FA oxidized. The 8-5′ (mainly in benzofuran form), 5-5′ and 8-O-4′ structures represented the 57, 40 and 3% of the total di-FA in AX, and the 66, 18, and 16% in AX gels, respectively.

### 3.2. Morphology and Microstructure of AXM

**Figure 4:**
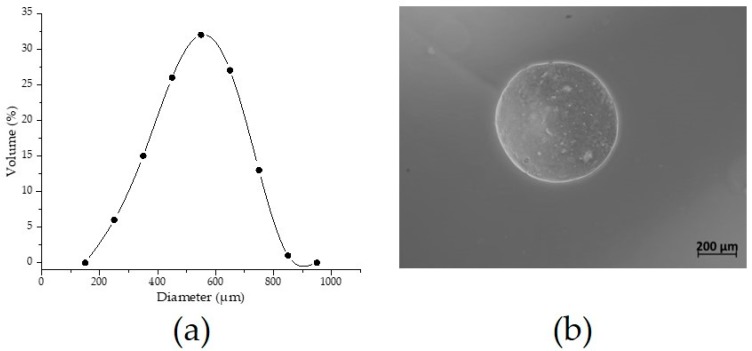
Characteristics of AXM (**a**) Diameter distribution; (**b**) Optical microscopy observation at 50× magnification.

**Figure 5:**
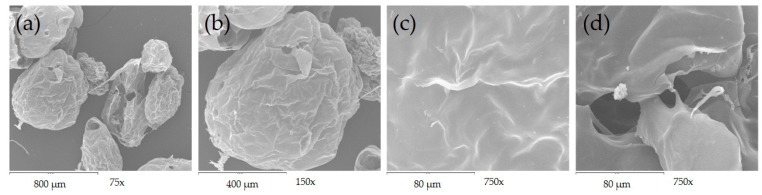
Scanning electron microscopy of lyophilized AXM at (**a**) 75×; (**b**) 150× and (**c**,**d**) 750×.

The AXM presented a size distribution ranging from 217 up to 775 µm, while the mean particle diameter value was found to be 533 ± 136 µm (Figure 4a). The optical micrographs of AXM showed a spherical shape and no aggregations were detected (Figure 4b). As shown in Figure 5, the scanning electron microscopy (SEM) images revealed that AXM exhibited a wrinkled, rough surface. No aggregates were observed, although some broken microspheres were identified (Figure 5a). The morphology of AXM appears as a three-dimensional and porous network, exhibiting some irregular cavities in its microstructure (Figure 5d).

### 3.3. Fourier Transform Infrared Spectroscopy

**Figure 6:**
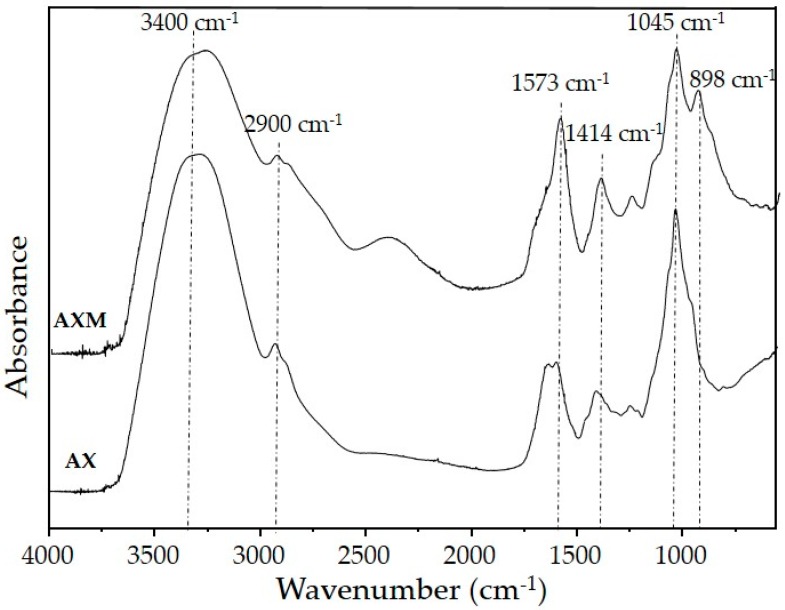
Fourier Transform Infrared Spectroscopy (FT-IR) spectra of AX and AXM.

The infrared analyses were performed in order to compare the structural characteristics of AX before and after the cross-linking process (Figure 6). Initially, the non-crosslinked AX spectrum was found to be similar to those previously reported for other AX [20,21,29]. Both spectra showed the absorbance region between 1200 and 900 cm^−1^, which is typical for arabinoxylan-type polysaccharides [50,51]. The absorption bands identified at 1045 and 898 cm^−1^ signals could be related to the antisymmetric C-O-C stretching mode of the glycosidic link and β-(1-4) linkages between the sugar units of the linear xylose backbone of AX [50,52]. The region from 3500 to 1800 cm^−1^ exhibits a broad stretching peak around 3400 cm^−1^ for the –OH group and a weak peak at 2900 cm^−1^ corresponding to the CH_2_ groups [53,54]. The loss of peak multiplicity in 1120–1000 cm^−1^ is a typical indication of a highly substituted AX, which is in accordance with the high A/X ratio (1.1) of the AX used in the present study [50]. The presence of the intense peaks at 1573 cm^−1^ and 1414 cm^−1^ can be observed in the AX spectrum after the cross-linking process (AXM). The absorption band at 1573 cm^−1^ has been related to the stretching of carboxylic anions indicating the formation of monoesters, which could be attributed to the aromatic skeletal vibrations in association with lignin [50]. In addition, an intense peak associated with the CO asymmetric stretching was detected at 1414 cm^−1^, indicating the increase of ester groups [43]. These modifications in the peaks suggest the cross-linking of AX chains during oxidative gelation induced by laccase of AXM.

### 3.4. Swelling and Structure

**Figure 7:**
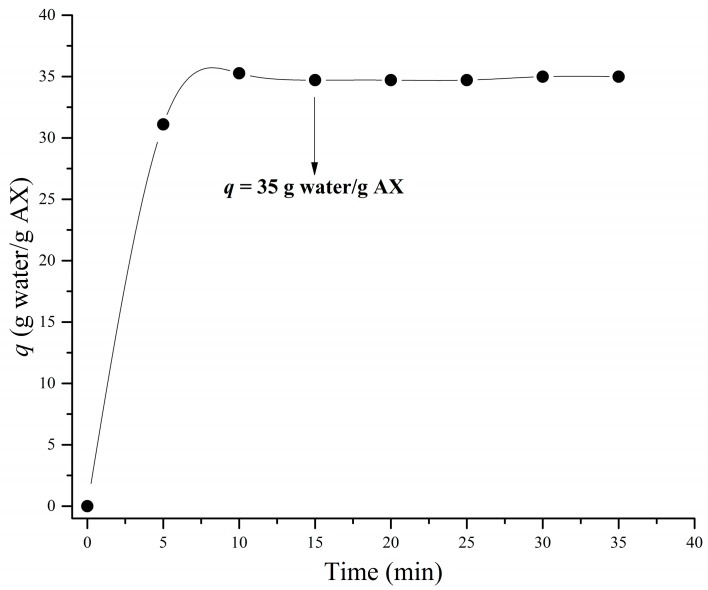
Swelling kinetic curve of AXM (2% w/v) in sodium azide solution (0.02% w/v) at 25 °C. Arrow indicates the *q* value at the equilibrium swelling.

The swelling behavior of AXM was monitored for 35 min at 25 °C (Figure 7). The equilibrium swelling of AXM was reached at 15 min and the swelling ratio (*q*) was 35 g water/g AX. The *q* value allows the calculation of the structural parameters of AXM. Table 2 presents the molecular weight between two cross-links (*M_c_*), cross-linking density (*ρ_c_*) and mesh size (*ξ*) of AXM. A higher *ξ* value (52 nm) and lower di-FA and tri-FA contents have been reported for maize AX gels using a higher polysaccharide concentration (4% w/v) [33]. In addition, a higher *ρ_c_* and lower *M_c_* values were obtained in the present work in comparison to those observed for wheat AX gels [55].

### 3.5. In Vitro Antioxidant Activity

The in vitro antioxidant capacity of AX gels and AX microspheres was previously evaluated only by the ABTS^+^ method [20,43]. In this study, three in vitro assays named ABTS^+^, DPPH, and FRAP were used in order to investigate the antioxidant capacity of AX and their gels. DPPH and ABTS^+^ methods are based on a similar principle. Both methods allow the determination of the antioxidant capacity through a mechanism which involves the transference of electron by the reducing agent (antioxidant) to the generated radical (DPPH or ABTS^+^) [42,56]. On the other hand, FRAP method measures the metal reducing power potential [50]. Table 3 shows the TEAC values of AX before and after the gelation process obtained by the ABTS^+^, DPPH, and FRAP methods. According to the results, a similar behavior in antioxidant capacity was observed for AX and AX gels using the three mentioned methods. Results showed that the cross-linking process reduced the antioxidant activity of AX by 61–64 % as observed in the ABTS^+^, DPPH, and FRAP methods.

### 3.6. Effect of AX and AXM on Cell Proliferation

**Figure 8:**
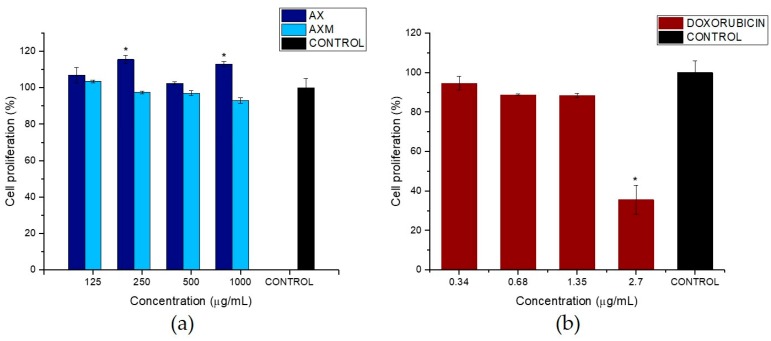
Effect of (**a**) AX and AXM and **(b**) doxorubicin on the cell proliferation of CCD 841 CoN. Cells were incubated with different concentrations of AX, AXM, and doxorubicin in cell culture medium for 48 h before cell proliferation was measured. Significant differences (*p* < 0.05) from dissolvent control are marked with asterisk.

**Figure 9:**
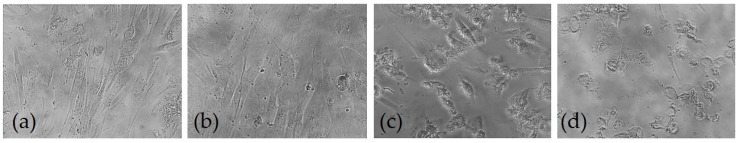
Optical micrographs of CCD 841 CoN cells. (**a**) Control, (**b)** treated with AX, (**c)** treated with AXM and (**d**) treated with doxorubicin for 24 h. Cells were treated with 1000 µg/mL of AX and AXM, and 2.7 µg/mL of doxorubicin. Magnification 200×.

In the present study, the potential toxic effect of AX and AXM on the proliferation of CCD 841 CoN cell line was evaluated using the colorimetric MTT method. Different concentrations of AX and AXM (125, 250, 500 and 1000 µg/mL) were prepared in order to evaluate the biocompability of AX and AXM with the human colon cell line CCD 841 CoN. Different concentrations of doxorubicin (0.34, 0.68, 1.35, 2.70 µg/mL) were also prepared and used as positive control. The results showed that AX did not have an evident effect on the proliferation of the human colon cell line (CCD 841 CoN) (Figure 8a). Indeed, an interesting observation was that morphology of treated cells with AX was similar to that exhibited by untreated cells and those treated with dissolvent control (H_2_O), independently of the concentration used. In a similar way, the AXM treatment showed no effect over proliferation and morphology of CCD 841 CoN cells (Figure 8 and Figure 9). A cell proliferation of over 96% was observed for all the concentrations used in the experiments, in comparison with dissolvent control. The cytotoxic agent doxorubicin was used as positive control in the cell proliferation assays. Figure 8b shows that doxorubicin exhibited antiproliferative activity on the CCD 841 CoN cell line. The cytotoxic agent was more effective at suppressing the cell proliferation at the higher concentration used in the experiment (2.7 µg/mL) and showed a dose-dependent response.

Figure 9 shows the morphology of CCD 841 CoN cells treated with AX and AXM at 1000 µg/mL and doxorubicin at 2.7 µg/mL for 24 h. The optical micrographs evidence that AX and AXM did not have any effect on the proliferation and morphology of CCD 841 CoN, which is in agreement with the results observed in the MTT assays. It can be observed that some of the CCD 841 CoN cells treated with AXM appear to be in suspension; this behavior could be related to the presence of AXM in the culture medium which may be decreasing the number of adhering cells. Conversely, an evident effect on the proliferation and morphology of the cells treated with the highest concentration of doxorubicin (2.7 µg/mL) for 24 h was observed. The images showed a significant decrease (>60%) on cell proliferation of CCD 841 CoN cells, in comparison to the untreated cells, as well as dissolvent control treated cells. In addition, the treatment with doxorubicin caused some morphological alterations on the cells, particularly an evident effect on the cell shape was observed.

## 4. Discussion

The kinetic of gelation of AX solution showed typical behavior for this polysaccharide, as has been observed for other maize AX induced by laccase [7,29,34,57]. During the formation of the gel, the storage modulus (G′) increased with time, while the loss modulus (G′′) decreased, which is related to the formation of covalent cross-links between the polysaccharide chains. The *t_g_* at 15 min indicates that at this point, AX presented a more gel-like structure than a viscous or liquid. After the *t_g_* point, the cross-links continued increasing until the gel was completely formed as evidenced by the maximum G′ value reached in the plateau region. Martínez-López et al. [7] reported a similar G′ value (285 Pa) using a higher polysaccharide concentration (4% w/v) in comparison to the concentration used in the present study. This difference could be attributed to the structural characteristics of the molecule (A/X ratio, Mw, FA), and particularly the FA content of AX used in this study, which was much higher than that used in previous study (0.25 µg/mg AX). The central role of the feruloylation degree of AX in the gel strength was confirmed previously [35]. The mechanical spectrum showed the typical behavior of a viscoelastic material, which evidenced the formation of the AX gel, as has been reported by previous studies [7,10,29].

The content of FA in AX mainly depends on the source of the polysaccharide, as well as the method and conditions used for its extraction. Lower content of FA has been identified in AX from other sources such as nejayote and wheat [16,52]. In contrast, Morales-Burgos et al. [34] and Mendez-Encinas et al. [29,57] obtained similar values (6.05–7.53 µg/mg AX) for maize bran AX under the same extraction conditions used in the present study. These authors used short time alkaline hydrolysis (30 min), as this condition preserves the FA content of the polysaccharide. In addition, similar amounts of di-FA and tri-FA have been previously detected in maize bran AX by Morales-Burgos et al. [34]. The amounts of di-FA and tri-FA detected in AX gels were similar to those reported for maize bran AX gels (1.24 and 0.07 µg/mg AX for di-FA and tri-FA, respectively) using a similar polysaccharide concentration [34]. Similarly to other studies, the amount of di-FA and tri-FA formed during gelation did not correspond to the loss of FA monomer oxidized. Indeed, ~35% of the initial FA content was oxidized, but only 85% was recovered as a combination of di-FA and tri-FA forms in AX gel. Such behavior appears to be related to the formation of superior ferulated cross-linking structures, as have been suggested by several authors [18,29,58]. The presence of the 8-5′ di-FA (benzofuran form) has been identified as the predominant structure in other maize and wheat AX gels induced by laccase and it is related to the presence of inter chain cross-links [29,34,58].

The use of different methods such as electrospraying and dropwise extrusion have permitted the fabrication of novel AX-based materials such as particles, microbeads, and microspheres [6,7,32]. Several factors including structural characteristics and concentration of the polysaccharide, the method and conditions of fabrication, among others, could influence the characteristics of the material. Particularly, coalescence and aggregation phenomena represent important drawbacks during nano and micro particles fabrication as they contribute to the size polydispersity of these materials [32]. In AX gelation, these phenomena strongly depend on the *t_g_* and also the FA content of the polysaccharide, as they are main features for the cross-linking process and consequently particle surface stabilization. Highly ferulated AX allow a faster gelation for lower *t_g_* which contribute to diminish the aggregation and coalescence of the particles produced. In this study, AXM were obtained by the coaxial electrospraying method and neither aggregation nor coalescence were observed during fabrication. AXM showed a similar size distribution (350 up to 798 µm) and mean diameter (531 µm) than those obtained for AX microspheres produced by the dropwise extrusion method [33]. Paz-Samaniego et al. [6] obtained electro sprayed core-shell particles with a higher average diameter (1.4 mm), which could be attributed to higher polysaccharide concentrations (4 and 10% v/w) used in the system and due to the insulin and bifidobacteria encapsulated in the particles. The structural characteristics of the polysaccharides, as well as the treatments used prior to the analyses impact on the morphology and microstructure of the material obtained [22]. Therefore, the presence of some broken AXM could be related to the freeze-drying process used prior to the SEM analyses. The freeze-drying process involves the formation of ice-crystals which can disrupt the structure of the material as has been reported previously in freeze-dried xylan-based microparticles [59].

The structural parameters of AXM can be calculated after the determination of its swelling degree. The evaluation of these parameters allows us to understand how the cross-linking process impacts the characteristics of the gels formed. Therefore, the knowledge of these characteristics may give important information to be considered for the possible applications of these materials. The *q* value indicates the amount of water that can be absorbed by the gel and it is considered an important factor in materials used for oral drug delivery [22]. In a previous study, maize bran AX microspheres reached a lower *q* value (18 g water/g AX) than AXM [33,43]. This difference could be related to the lower polysaccharide concentration used in the present study (2% w/v) in comparison to the higher concentration used in that previous work (4% w/v). It has been stated that higher *q* values are related to a weaker gel structure and high water absorption. A lower polysaccharide concentration leads to the presence of longer uncross-linked AX chains sections, which expand easily and result in a weaker structure of the gel increasing the water uptake [60]. Other studies have reported similar *q* values (28–43 g water/g AX) for maize AX cylindrical hydrogels [21,57]. Nevertheless, the time to reach equilibrium swelling (15 min) in AXM was very short in comparison to that reported for cylindrical hydrogels (4–12 h) [21,57]. Such behavior can be explained as a consequence of the higher surface area in AXM, which facilitates the uptake of water into the gel and decreases the time to reach the equilibrium swelling [33]. In the present study, the high content of FA (5.45 µg/mg AX) of the AX promoted the formation of higher amounts of di-FA and tri-FA, as well as other possible superior structures and/or physical interactions between AX chain [30]. The increase of cross-linking structures resulted in a gel with a more compact microstructure, a high cross-linking density and smaller mesh size. These types of structures are desirable for use in encapsulation and controlled-release systems.

The results of the in vitro antioxidant capacity assays demonstrated that the gelation of AX leads to a decrease in this property. This behavior has been previously observed for AX gels and AX microspheres [20,43]. The decrease in the antioxidant activity of AX gels could be related to the cross-linking process involving FA oxidation and its subsequent coupling for the formation of the di-FA and tri-FA. In addition, the lower movement of the polysaccharide chains by the rigidity of the gel (293 Pa, Figure 3a) could also affect the antioxidant activity [20,43]. Herrrera-Balandrano et al. [25] obtained 21, 32, and 58 µmol TEAC/g sample for nixtamalized maize bran AX in the ABTS^+^, DPPH, and FRAP methods, respectively. In the present study, the higher antioxidant activity of AX obtained by the ABTS^+^ method could be attributed to the higher content of FA (5 µg/mg AX) of the polysaccharide in comparison to the lower content used in that study (2 µg/mg AX). Antioxidant capacity of AX has been widely related to the concentration of hydroxycinnamic acid acids, mainly the FA content [44,61,62]. Particularly, a positive correlation between the FA content and the ABTS^+^ and DPPH antioxidant activity was found by Malunga and Beta [44]. On the other hand, the lower value obtained in the metal reducing potential of AX could be associated with the structural characteristics of the molecule, specifically the total phenolic compounds and protein content. The presence of protein in water soluble AX from wheat appears to be associated with a decrease in the antioxidant capacity, while a higher content of total phenolic compounds increases such property [50]. Therefore, it could be possible that the lower protein content and the high content of total phenolic compounds in the AX investigated by Herrera-Balandrano et al. [25] contributed to some extent to its antioxidant capacity. However, the observations show that even after the gelation process, AX exhibited antioxidant activity showing higher TEAC values than those previously reported for AX gels and AX microspheres (11 and 13.24 µmol TEAC/g) using the ABTS^+^ assay [20,43]. The antioxidant capacity of the AX gels could be related to the high content of remnant FA (3.52 µg/mg AX) after the gelation process (Table 3). A considerable amount of not-oxidized FA can be detected in AX gels after the cross-linking process as observed by several authors [10,29]. Such residual FA may have the ability to scavenge the free radicals and act as antioxidant agent. Moreover, it has been suggested that the presence and amount of tri-FA in AX contributes to the scavenging capacity of the polysaccharide [63]. The authors of such study indicate that the antioxidant capacity of the trimer is related to its structural characteristics, as it is composed of three FA molecules, which provide more –OH groups with hydrogen donor capacity. According to this statement, it is possible that the higher content of di-FA and tri-FA detected in AX gels could also contribute to the antioxidant capacity of the gels. However, the higher contribution to this property may be associated with the high amount of residual FA detected in AX gels. The results obtained in these experiments demonstrate that AX exhibit antioxidant capacity even after the cross-linking process and could be excellent candidates for their use as matrices with antioxidant activity.

In recent years, some studies have suggested that natural antioxidants may act as prooxidants, which generate free radicals and cause DNA damage and mutagenesis [64]. Phenolic compounds are well-known antioxidants because they can inhibit reactive oxygen species (ROS). However, several reports have shown that these compounds can also act as prooxidants under certain conditions, including high pH, high concentration, and mainly in the presence of transition metals such as Fe and Cu [65,66]. Phenolic compounds exert their prooxidant activity by the formation of phenoxy radicals or redox complexes with transition metal ions [64]. It should be noted that the prooxidant activity of natural antioxidants has been mainly evaluated using in vitro conditions which do not necessarily translate to in vivo systems. In addition, it has been stated that the prooxidant activity of natural antioxidants can increase the ROS quantity to levels that are cytotoxic for cancer cells but not for normal cells. Cancer cells have greater metabolic activity and higher concentrations of copper ions in comparison to normal cells, which make them more susceptible to the prooxidant activity of antioxidants [64]. Therefore, some natural antioxidants could be used to treat cancer.

To date, there is no evidence related to the prooxidant activity of neither AX nor their gels. Moreover, the amount of phenolic acids linked to AX is very low and represents <1% of the total polysaccharide composition [61]. Considering the prooxidant activity of natural antioxidants is related to high doses, the low phenolic acids content in AX and their gels could not have an impact on such activity. However, the prooxidant activity of AX and their gels under in vitro and in vivo conditions should be further investigated.

The MTT assays have demonstrated that both AX and AXM did not inhibit the proliferation of the human colon cell line CCD 841 CoN (Figure 8a). Wang et al. [57] observed that MCF-7 cells treated with AX had better growth due to the fair nutritive value of AX. A similar behavior was reported by Samuelsen et al. [46] where the treatment of AX from barley (0.5–3 mg/mL AX) had no significant effect on the proliferation of two human colon cancer cell lines, HT-29 and Caco-2. Moreover, in a previous study, AX foams prepared by peroxidase/H_2_O_2_ enzymatic cross-linking reaction did not have a cytotoxic effect on NIH3T3 fibroblasts [67]. In that study, the cell viability values (~96%) were close to those observed in the non-cancerous cell line (CCD 841 CoN) treated with AXM, which was evaluated in the present study. Moreover, Rodrigues et al. [59] found that xylan was biocompatible with HeLa cells in the range of 4.1–12.4 mg/mL due to the non-toxic and highly safe characteristics of the biopolymer. The observations found in the present study demonstrate that AX and AXM exhibit good biocompatibility and do not have a cytotoxic effect on the normal human colon cell line CCD 841 CoN. These findings could allow for the use of the AXM as a novel biocompatible material for pharmaceutical purposes. On the other hand, the treatment with the cytotoxic drug doxorubicin showed a clear effect on the proliferation of CCD 841 CoN cells (Figure 8b). The results obtained in the cell proliferation assays are in accordance with observations of the morphological characteristics of the cells exposed to the different treatments (Figure 9). The cytotoxic effect of doxorubicin was evidenced by the notable morphological alterations observed in the cells. Doxorubicin is a very potent antineoplastic agent used for the treatment of several types of cancer. The treatment of H9c2 cells with doxorubicin causes morphological alterations in mitochondrial, nuclear, and fibrous protein structures, which are dependent on drug concentration [68].

## 5. Conclusions

In this work, AXM were prepared by oxidative cross-linking using the coaxial electrospraying method. AXM were spherical and exhibited a heterogeneous and porous structure. The cross-linking of AX chains during the gelation process was confirmed by FT-IR analysis. The ABTS^+^, DPPH, and FRAP assays showed that AX exhibit antioxidant activity even after the gelation process. Moreover, the cytotoxicity assays demonstrated that AX and AXM do not inhibit the proliferation of the human colon cell line, CCD 841 CoN (non-cancerous). These findings suggest the biocompatibility of AX and AXM with normal human colon cells. The results indicate that AXM could be used as promising biocompatible materials with antioxidant activity.

## Figures and Tables

**Table 1:** Ferulic acid (FA), dimers of FA (di-FA) and trimers of FA (tri-FA) contents in AX before and after 1 h gelation.

Time	FA	di-FA	tri-FA	^a^ FA Oxidized	^b^ FA Recovered
(min)	(µg/mg AX)			(%)	
0	5.45 ± 0.09	0.35 ± 0.07	0.03 ± 0.00	-	-
60	3.52 ± 0.26	1.61 ± 0.14	0.04 ± 0.01	35 ± 5	85 ± 1

^a^ After 1 h of AX solution laccase exposure at 24 °C. ^b^ Calculated from the percentage of oxidized FA recovered as di-FA + tri-FA in the AX gel.

**Table 2:** Structural parameters of AX-based particles (AXM) (2% w/v) after 1 h gelation.

Parameter	Value
*q*^a^ (g water/g AX)	35 ± 1.50
*M_c_*^b^ × 10^3^ (g mol^−1^)	50 ± 0.10
*ρ_c_*^c^ × 10^−6^ (mol/cm^3^)	47 ± 0.01
*ξ ^d^* (nm)	27 ± 3.00

^a^ Swelling degree; ^b^ Molecular weight between two cross-links; ^c^ Cross-linking density; ^d^ Mesh size. Values are reported as means ± standard deviation of three experiments.

**Table 3:** Antioxidant activity of AX before and after gelation.

	Antioxidant Activity ^a^ (µmol TEAC/g)		
Sample	ABTS^+^	DPPH	FRAP
Uncross-linked AX	68.05 ± 0.53	32.23 ± 0.50	48.41 ± 1.07
Cross-linked AX	26.02 ± 3.82	12.58 ± 0.45	16.83 ± 0.83

^a^ TEAC, in µmol/g AX or AX gel. All values are means ± standard deviation of duplicate.
